# Impact of tumour size on axillary involvement and distant dissemination in breast cancer

**DOI:** 10.1038/sj.bjc.6605221

**Published:** 2009-08-18

**Authors:** S Koscielny, R Arriagada, J Adolfsson, T Fornander, J Bergh

**Affiliations:** 1 Department of Clinical and Translational Research, Institut Gustave-Roussy, Villejuif, France; 2 Unit of Cancer Epidemiology (Unit 605) National Institute of Health and Medical Research, Villejuif, France; 3 Department of Oncology-Pathology and Cancer Center Karolinska, Karolinska University Hospital and Karolinska Institutet, Stockholm, Sweden; 4 Stockholm and Gotland Oncologic Centre, Karolinska Hospital, Stockholm, Sweden

**Keywords:** breast cancer, prognosis, tumour size, parametric models

## Abstract

**Background:**

Tumour size and nodal involvement are the two main prognostic factors in breast cancer (BC). Their impact on the natural history of BC is not fully captured by analyses that ignore their quantitative nature.

**Method:**

Data pertaining to 18 159 patients treated with primary surgery: 3661 at the Institut Gustave-Roussy (IGR, France) between 1954 and 1983, and 14 498 in the breast cancer registry in the Stockholm–Gotland Health Care region (SG, Sweden) between 1976 and 1999, were collected. The risks of distant metastases (DMs) and of nodal involvement were analysed according to tumour size with parametric models.

**Results:**

Using SG 1976–1990 as the reference group, relative risks (RRs) for DM were equal to 1.42 (95% CI: 1.29–1.56; *P*<10^−10^) in IGR and 0.61 (95% CI: 0.55–0.67; *P*<10^−10^) in SG 1991–1999. Differences in tumour size explained the increased risk in IGR (RR adjusted for tumour size 1.09; 95% CI: 0.99–1.20; *P*=0.07), but not the decreased risk in SG 1991–1999 (adjusted RR: 0.63; 95% CI: 0.57–0.69; *P*<10^−10^). The relationship between tumour size and DM risk changed significantly during the 1990s.

**Conclusion:**

Early diagnosis is sufficient to explain differences in the prognosis before 1990. After 1990, the use of adjuvant systemic therapies is the main reason for the reduction in DM.

In developed countries, the incidence of breast cancer increased regularly from 1975 to 2000 (Parkin *et al*, 2001). A decrease in mortality was first observed in the United States during the 1990s (Ries *et al*, 2004). Recent publications by the Cancer Intervention and Surveillance Modeling Network (CISNET) attributed the decrease in case fatality to screening and adjuvant treatments (hormone and chemotherapy) (Berry *et al*, 2005; CISNET, 2006). The conclusions were supported by seven different models using analytical or simulation techniques and including from 6 to 40 parameters. Several models used the decreased mortality observed in clinical trials (EBCTCG 1998a, 1998b) as model inputs to estimate the effect of therapies on mortality.

In order to extend the work of the CISNET, we chose a simple approach based on the most relevant prognostic parameters: tumour size and axillary lymph node involvement. These parameters are the cornerstones of the TNM (tumour, the lymph nodes and metastasis) system and of new prognostic tools such as Adjuvant! (http://www.adjuvantonline.com) (Ravdin *et al*, 2001). We focused on distant metastases instead of mortality in order to avoid any problem related to the definition of breast cancer mortality or to changes in mortality patterns in a population. We used tumour size as a quantitative variable to maximise the use of the information. Our strategy is based on analyses of the relationship between tumour size and the probability of distant metastasis (Koscielny *et al*, 1984). This strategy was used during the 1980s to build a simulation model that predicted a roughly 30% decrease in the incidence of metastases with screening (Koscielny *et al*, 1985). These results, which were among the theoretical arguments used to justify screening (Tubiana and Koscielny, 1990), are actually in keeping with the results of screening trials (Nyström *et al*, 2002).

As the study period spans about 50 years, we also investigated the effect of the year of treatment and, consequently, of the effect of changes in management strategies (early detection, introduction of organised mass screening, adjuvant endocrine or cytotoxic therapies) on the relationship between tumour size and the risks of nodal involvement and metastasis.

## Materials and methods

### Institut Gustave-Roussy database

This database includes 7688 consecutive patients with breast carcinoma treated at the Institut Gustave-Roussy (IGR; France) between 1 January 1954 and 31 December 1983. The database was updated every 2 years and last updated in 2003–2005.

### Stockholm–Gotland Health Care region breast cancer registry

The Stockholm–Gotland Health Care region (SG) registry (Sweden) covers a population of about 1 million women (946 784 in 1999), and around 1000 new breast cancer cases are now registered annually. Follow-up is continuously updated. During the period spanning from 12 April 1976 to 31 December 1999, 21 700 breast cancer patients were included. The last follow-up date was 1 April 2005. The Stockholm mammography screening programme was started in 1989, inviting women between 50 and 69 years of age to have a mammography at 2-year intervals (Törnberg *et al*, 2005).

### Patient selection

The only available and reliable measurement of tumour size in the two databases was the largest diameter measured on the surgical specimen in patients who had undergone primary surgery. To use similar information in the two databases, we selected patients with invasive breast cancer who first underwent surgery and for whom tumour size and the number of histologically involved nodes were available. The tumour grade and hormone receptors were not included in the analyses. Patients with detectable metastases at diagnosis or patients who had previously been treated for another cancer were excluded. Thus, 18 159 out of 29 388 patients present in the two databases were analysed.

### Relationship between tumour volume and the proportion of patients with events

Logistic, complementary log–log and probit regression models were used to analyse the relationship between tumour size (a quantitative variable) and qualitative data (axillary node involvement, metastasis occurrence). The logistic and complementary log–log regressions (or Cox's model when time to event was used as a continuous variable) led, respectively, to odds ratio and relative risk estimates, which are useful for assessing risks but not for understanding the mechanisms or the natural history of the disease. Probit regressions were used to estimate tolerance distributions, for example, distributions of tumour volume required to obtain a given proportion of patients with involved nodes, or a given proportion of patients with detectable metastases within 5 years of treatment.

The tumour volume was calculated from the largest tumour diameter assuming spherical lesions. In the regression models, the quantitative variable used for tumour size was the logarithm of tumour volume. We analysed two end points: the number of involved axillary lymph nodes (used as an ordinal variable) and the occurrence of distant metastases during the 5 years after treatment.

Axillary nodal involvement was analysed through regressions between tumour size and the number of positive axillary lymph nodes. The number of sampled nodes was introduced as a covariate with five categories (1–6, 7–9, 10–14, 15+ and unknown).

The relationship between tumour size and metastasis occurrence was analysed after grouping tumours by diameter in 1-cm increments. We estimated the 5-year cumulated proportion of patients with metastases, and the geometric mean tumour size in each group.

We used the Kaplan–Meier method to estimate the proportions of patients with distant metastases. Only distant metastases registered as first events were counted as events. Patients who experienced a competing event first were censored at the date of the first event. Deaths were never counted as events: distant metastases followed by deaths were counted as an event at the time of the first distant metastasis; patients dying without distant metastases were censored at death. Local recurrences that occurred first were not counted as events; patients were censored at the time of the local recurrence, and we implicitly assumed that their risk of distant metastases was similar to that of women who had not experienced a local recurrence.

### Dissemination patterns according to the patient's origin (IGR *vs* SG) and the period of treatment

Patients were distributed into three groups as follows (Table 1): (1) all patients from IGR (IGR 1954–1983); (2) patients treated in the Stockholm–Gotland region between 1976 and 1990 (SG 1976–1990); and (3) patients treated in the Stockholm–Gotland region after 1990 (SG 1991–1999). Lymph node involvement and the 5-year risk of metastases were analysed with respect to tumour size, assuming a relationship with identical slopes in the three groups.

The tumour volume corresponding to a 50% 5-year risk of metastases was calculated in each cohort. The number of involved nodes was analysed as a function of tumour size, the number of sampled nodes and the cohort. The tumour volume corresponding to 50% of tumours with involved nodes was calculated in each cohort (assuming a number of sampled nodes of between 7 and 9) and it was used as an indicator of the tumour's propensity to invade axillary lymph nodes.

### Evolution of dissemination patterns according to the year of surgery

The total population (18 159 patients) was divided into 18 consecutive cohorts of patients (17 first cohorts including 1000 patients and the last cohort including 1159 patients) with no distinction between IGR and SG patients. The relationship between tumour size and the proportion of patients with metastases at 5 years was analysed with probit regression, assuming similar slopes in all cohorts.

### Statistical tests

The distributions of qualitative variables were compared with *χ*^2^ tests and the distributions of quantitative variables with non-parametric Kruskall–Wallis tests. The proportions of patients free of distant metastases according to the time since treatment were compared with log-rank tests. The statistical significance of the regression parameters was assessed with Wald tests. All the analyses were carried out using Statistical Analysis System (version 8.2, SAS, Cary, NC, USA) software.

### Role of the funding source

This study was carried out by a collaboration between the Institut Gustave-Roussy (Villejuif, France), the Karolinska Institutet (Stockholm, Sweden) and the Stockholm–Gotland Breast Cancer Group. The two institutes funded the sabbatical periods spent by SK in Stockholm.

## Results

The three patient groups differed significantly for almost all study parameters (Table 1). Patients treated at IGR were about 6 years younger and consequently more frequently pre-menopausal than those from the Stockholm area. The major differences in tumour characteristics among the three groups were with regard to tumour size and the proportion of node-positive tumours. The most important therapeutic change was the major increase in the use of adjuvant endocrine therapy (mainly tamoxifen in postmenopausal women) from 23% before 1991 to 72% after 1990 (Table 1). Adjuvant chemotherapy, mostly cyclophosphamide methotrexate fluorouracil (CMF), was also used with ever-increasing frequency during the nineties.

### Incidence of distant metastases

**Figure 1:**
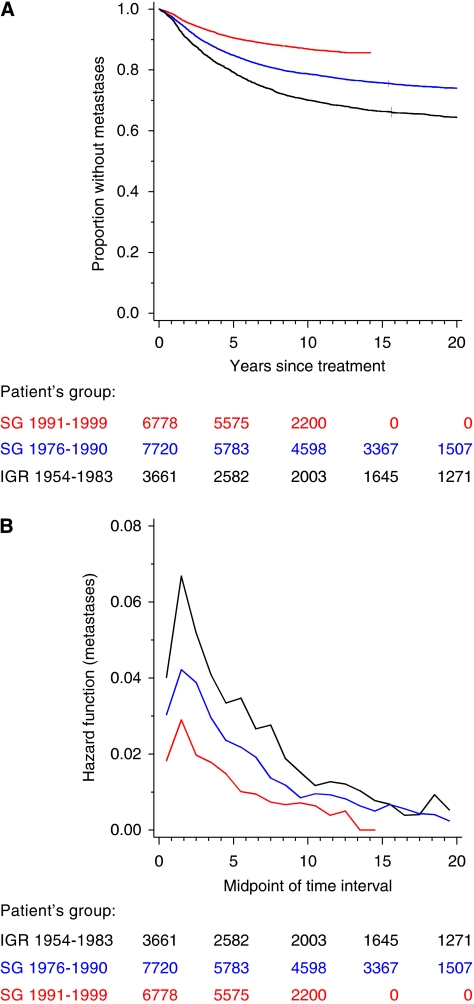
Distant metastasis-free rates (**A**) and corresponding hazard functions (**B**) according to patient groups.

The incidence of distant metastases according to time since treatment (Figure 1A) differed significantly between the three groups (*P*<10^−10^). The hazard functions for distant metastases were unimodal and the hazards reached a maximum during the second 1-year interval after treatment (Figure 1B). The hazards of metastases were roughly proportional between the three groups. With SG 1976–1990 as the reference group, the relative risks were equal to 1.42 (95% CI: 1.29–1.56; *P*<10^−10^) in IGR 1954–1983 and 0.61 (95% CI: 0.55–0.67; *P*<10^−10^) in SG 1991–1999.

All the regression models correctly fitted the relationships between tumour volume and risk of metastases; the differences in the adjustment to the data were not sufficient to exclude a particular model (data not shown). The relationships between tumour volume and the 5-year risk of distant metastases were not different between IGR 1954–1983 and SG 1976–1990 (Table 2a). Smaller tumour sizes in the Stockholm–Gotland region were sufficient to explain the difference in the prognosis between the two groups. The 5-year risks of distant metastases adjusted for tumour size were not significantly different (16.2 *vs* 14.8% for an 18-mm tumour) between the two groups. Similarly, the odds ratio (logistic model) and relative risks (Cox's model) for distant metastases were not different from 1 when adjusted for tumour size used as a continuous covariate. It should be noted that when tumour size was categorised with a simple cutoff at 3 cm, the odds ratio and relative risk became significantly different from 1 (last row of Table 2a).

In contrast, the decrease in the 5-year risk of distant metastases between SG 1976–1990 and SG 1991–1999 was not explained by differences in tumour size. In the three regression models, the relationship between tumour size and the probability of distant dissemination changed significantly between the two periods in the Stockholm material (*P*<10^−10^). The logistic regression and Cox models adjusted for tumour size were also consistent with a highly significant difference between the two groups (*P*<10^−10^). The tumour volume required for a 50% rate of metastases at 5 years was multiplied by >3 in SG 1991–1999 (>900 ml) compared with the other two groups (<300 ml).

### Nodal involvement

With SG 1976–1990 as the reference group, the risk of nodal involvement was significantly higher for IGR 1954–1983 (*P*<10^−10^), but was not different for SG 1991–1999 (*P*=0.60) (Table 2b). The tumour volume corresponding to 50% of patients with nodal involvement during the first Stockholm period was significantly larger from that in IGR 1954–1983 (*P*=0.03) and smaller than the volume estimated for the second Stockholm period (*P*=0.01). However, even if the differences concerning axillary node involvement as a function of tumour size were statistically significant, their clinical importance is limited: the difference in the risks of nodal involvement adjusted for tumour size (estimated for an 18-mm tumour diameter) was only 2% (*P*=0.01) between SG 1976–1990 and SG 1991–1999. Similar results were obtained when logistic regression was applied for axillary node involvement.

### Evolution of metastatic and nodal dissemination patterns according to the year of surgery

**Figure 2:**
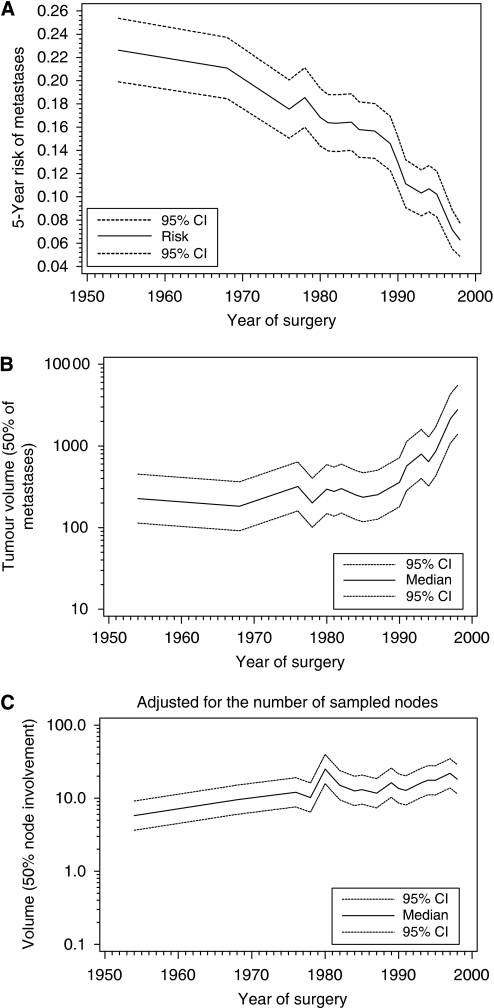
Variations according to the year of surgery for the whole population. (**A**) The 5-year proportion of patients with metastases decreases regularly with the year of surgery from more than 20% in the 1950–1960s to <8% in 1999. (**B**) The tumour volume corresponding to 50% of patients with distant metastases during the first 5 years remains stable up to the beginning of the 1990s. During this period, differences in tumour size at treatment are sufficient to explain the decrease in the risk of metastases. After 1990, the tumour volume corresponding to 50% of patients with metastases increased rapidly from about 200 ml in 1990 to 2000 ml in 1999. During this period, the amount of tumour necessary for metastatic dissemination apparently increased. The reason is either a reduced metastatic potential of the tumours or a decreased viability of the metastases. (**C**) There was no marked change in the year of treatment in tumour volume corresponding to 50% of patients with involved axillary nodes. The capacity of tumours to involve lymph nodes was almost unchanged from 1954 to 1999. This observation contradicts the hypothesis of a reduction in the metastatic potential of tumours after 1990. Curves are displayed with the 95% confidence intervals.

The 5-year probability of metastases decreased regularly according to the year of surgery (Figure 2A). Overall, the probability of metastases was divided by 3 between 1954 and 1999. The tumour volume resulting in 50% of patients with distant metastases at 5 years (Figure 2B) did not vary significantly between 1954 and the beginning of the 1990s and increased markedly afterwards, when it was multiplied by a factor of 10 (from about 200 ml before 1990 to more than 2000 ml in 1999). This apparent decrease in the capacity of tumours to generate detectable metastases in the five post-treatment years contrasts with a stable ability to invade axillary lymph nodes (Figure 2C).

### Comparison between IGR and SG over the 1976–1983 period

We also performed a comparison between IGR and SG that was limited to the 5057 patients treated during the 1976–1983 period, which is common to the two centres: 1756 patients in IGR and 3301 in SG. The differences/similarities between these two populations were the same as that observed in the broader comparisons between IGR 1954–1983 and SG 1976–1989. The tumour size, the proportion of patients with involved nodes and the 5-year proportion of patients with metastases were significantly larger in IGR 1954–1983 (*P*<10^−10^, *P*<10^−10^ and *P*<10^−5^, respectively), whereas the relationships between tumour size and axillary lymph node positivity and 5-year proportion of patients with metastases were not significantly different. All the conclusions regarding comparisons between IGR 1954–1983 and SG 1976–1989 are also valid for those between IGR and SG that were restricted to patients treated during the 1976–1983 period.

## Discussion

This study included patients treated in two European countries, over a 50-year period. The most striking result is the regular decrease in the risk of developing distant metastases according to the year of surgery. For a patient treated in 1999, the risk of metastases was about one-third than that of a patient treated in the 1960s (*P*<10^−10^). The issue was to determine to what extent this variation was attributable to differences in tumour malignity, or to earlier diagnosis or to better treatment strategies. Our main findings were (a) the constancy of the relationship between tumour size and nodal involvement, (b) no significant differences in the relationship between tumour size and the 5-year risk of distant metastases for IGR 1954–1983 and SG 1976–1990 and (c) a change in this relationship after 1990 in the Stockholm database.

### Constancy of the relationship between tumour volume and nodal involvement

Nodal involvement was used as a measure of the tumour's ability to spread (Carter *et al*, 1989; Koscielny *et al*, 1989; Michaelson *et al*, 2003a). The tumour volume required to obtain 50% of patients with involved nodes was deduced from nodal involvement and tumour size, two parameters that are available before the use of any adjuvant treatment. We considered that this volume could not be affected by the use of adjuvant treatments and was a measure of the intrinsic malignant capacity. Our observations over 50 years suggest a remarkable stability of the malignant capacity of tumours: the tumour volume required for nodal involvement increased by <3% yearly over the 50 years covered by the study (Figure 2C). These data strongly suggest that the decrease in breast cancer mortality observed in many countries is not due to a decreased malignant capacity.

### Similar relationships between tumour volume and 5-year risk of distant metastases for IGR 1954–1983 and SG 1976–1990

The risk of metastases was significantly higher in IGR 1954–1983 than in SG 1976–1990 (RR=1.42; 95% CI: 1.29–1.56; *P*<10^−10^). However, the relationship between tumour volume and risk of metastases was not significantly different between the two groups (*P*=0.07), nor was the relationship between tumour volume and nodal involvement (*P*=0.03). When adjusted for tumour size, the relative risk of metastases was not significantly different between the two groups (RR=1.09; 95% CI: 0.99–1.20; *P*=0.07). This finding implies that differences in the initial tumour volume, related to early diagnosis in SG, explain most of the difference in prognosis between the two groups of patients.

### Early diagnosis and screening

Surprisingly, the highly significant differences in tumour size at treatment were not related to the screening programmes that took place in the SG region. The Stockholm trial began in 1981 (Frisell *et al*, 1997), covering about 25% of the population of the Stockholm–Gotland region and was converted into a service screening programme covering the entire region in 1989 (Törnberg *et al*, 2006). The superiority of the service screening programme over the initial screening trial is not perceptible in our data, as the geometric mean tumour diameter decreased by only 2 mm after the start of the service screening programme. Regardless of the reasons for the patient participation in screening programmes, our data show that early diagnosis has a real impact on the reduction of breast cancer mortality. Our data support screening because it would not be ethical to be perfectly aware that early diagnosis reduces cancer mortality without providing a tool for early tumour detection. Before sending out negative messages, screening detractors should consider countries where breast cancer mortality is related to very late detection of tumours, and not only to slightly earlier treatment due to screening in countries where early detection is already a reality.

### Tumour stage *vs* tumour size

We found only one study evaluating the impact of early diagnosis using quantitative estimates of tumour size (Michaelson *et al*, 2003b). Many other studies used stage (or ranges of tumour size) instead of the actual tumour size. Their results should be regarded with caution because they are subject to a stage migration bias. This bias results from better detection of tumours, which increases the prevalence of good prognosis tumours (migration from the set of undetectable to the set of good prognosis tumours). As these additional tumours have a better prognosis than the previous group of good prognosis tumours, the overall prognosis of the latter group is improved. The first consequence of stage migration is that it alters the prognostic meaning of the stage. The way to overcome this bias is to use quantitative data instead of the qualitative stage. To illustrate this (Table 2a), the 5-year relative risk of metastasis comparing IGR 1954–1983 with SG 1984–1990, adjusted for the tumour size, was equal to 1.09 (95% CI: 0.99–1.20; *P*=0.07), whereas the relative risk adjusted for the tumour size category (cutoff at 3 cm) was equal to 1.29 (95% CI: 1.17–1.41; *P*=10^−7^). This example emphasises the importance of analyses using quantitative data and suggests that some recent results, such as a significant independent effect of mammography, observed by Shen *et al* (2005) might be, at least partially, a consequence of stage migration.

### Improvement of prognosis after 1990

The difference between the two consecutive groups of patients from the Stockholm–Gotland region (SG 1976–1990 and SG 1991–1999) was not explained by differences in the initial tumour volume, or by a change in the intrinsic malignity of tumours. This difference can only be attributed to a decrease in the viability of metastases due to adjuvant therapy. The more frequent use of endocrine therapy – mostly tamoxifen– which increased from <20% to about 80% in the few years after 1990 (Kemetli *et al*, 2009) is the most plausible single explanation for our observations. Further analyses are needed to evaluate the real impact of adjuvant chemotherapy at the population level and the impact of changes in the treatment strategy such as the prolonged duration of tamoxifen administration (in 1996), the use of anthracyclines (1999) and, more recently, the introduction of taxanes. A detailed study of these adjuvant treatments would necessitate extending the study population to patients treated after 1999.

In conclusion, our results support the conclusions of the CISNET group and extend their findings. In our clinical series, early diagnosis is sufficient to explain the differences in the prognosis before 1990. After 1990, the generalisation of adjuvant systemic therapy, mostly tamoxifen, is the main reason for improved disease control and better use of adjuvant chemotherapy is likely to improve outcomes further.

## Figures and Tables

**Table 1:** Patient and treatment characteristics

	**IGR**	**Stockholm (SG)**	
**Period**	**1954–1983**	**1976–1990**	**1991–1999**
Number of patients	3661	6778	7720
Age at diagnosis (years)	54 (5 missing data)	61	58
Pre-menopausal (%)	40.9	26.4	28.5
Geometric mean tumour diameter (mm)	22.0	18.0	16.0
Median number of sampled nodes	15.0	7.0	9.0
Number of patients with missing data	5	1361	47
Patients with node-positive disease (%)	56.3	34.6	34.3
Total mastectomy (%)	78	81	46
Postoperative radiotherapy (%)	61	35	59
Adjuvant chemotherapy (%)	2	9	20
Adjuvant endocrine therapy (%)^a^	23	23	72

IGR=Institut Gustave-Roussy; SG=Stockholm-Gotland Health Care region.

a At the IGR 1954–1983, adjuvant endocrine therapy was ovarian ablation in pre-menopausal patients. In Stockholm, it was mostly tamoxifen in postmenopausal patients.

No *P*-values are given because, due to the high number of patients, virtually all the differences are statistically significant.

**Table 2a:** Analysis of 5-year distant metastases according to the origin of the patients and period of surgery

**Total (*n*=18159 patients)**	**IGR 1954–1983 (3661)**	**SG 1976–1990 (6778)**	**SG 1991–1999 (7720)**	*P*-values*
Geometric mean tumour diameter (mm)	22.0	18.0	16.0	<10^−10^/<10^−10^
Absolute risk of distant metastases at 5 years (95% CI)	20.8% (19.5–22.2)	15.2% (14.4–16.0)	9.5% (8.8–10.2)	<10^−10^/<10^−10^
Relative risks of metastases (95% CI)	1.42 (1.29–1.56)	1 (reference)	0.61 (0.55–0.67)	<10^−10^/<10^−10^
Probit analysis: estimated tumour volume (ml) corresponding to 50% of patients with metastases at 5 years (diameter mm)	237 ml (77 mm)	305 ml (83 mm)	985 ml (123 mm)	0.07/<10^−10^
Probability of metastases for a 18-mm tumour (+) (%)	16.2	14.8	9.5	
Odds ratio (logistic regression) adjusted on continuous tumour size (95% CI)	1.11 (0.99–1.23)	1 (Reference)	0.60 (0.54–0.67)	0.07/<10^−10^
Relative risks (Cox's model) adjusted on continuous tumour size (95% CI)	1.09 (0.99–1.20)	1 (Reference)	0.63 (0.57–0.69)	0.07/<10^−10^
Relative risks (Cox's model) adjusted on tumour size category (<30 *vs* ⩾30 mm) (95% CI)	1.29 (1.17–1.41)	1 (Reference)	0.61 (0.55–0.67)	10^−7^/<10^−10^

IGR=Institut Gustave-Roussy; SG=Stockholm-Gotland Health Care region.

^*^P-values: the 1st *P*-value refers to the IGR 1954–1983 *vs* SG 1976–1990 comparison, the 2nd to SG 1976–1990 *vs* SG 1991–1999.

(+) 18 mm is the geometric mean tumour diameter in the overall population.

**Table 2b:** Analysis of nodal involvement according to the origin of the patients and period of surgery

**Total (*n*=18 159 patients)**	**IGR 1954–1983 (*n*=3661)**	**SG 1976–1990 (*n*=6778)**	**SG 1991–1999 (*n*=7720)**	*P*-values*
Geometric mean tumour diameter (mm)	22.0	18.0	16.0	<10^−10^/<10^−10^
Patients with node positive disease (%)	56.3	34.6	34.3	<10^−10^/0.73
Probit analysis: tumour volume (ml) corresponding to 50% of patients with involved nodes (diameter mm)	10.7 ml (27.4 mm)	13.8 ml (29.8 mm)	17.7 ml (32.3 mm)	0.03/0.01
Probability of node involvement for an 18-mm tumour (+) (%)	37.9	35.6	33.4	
Odds ratio (logistic regression), adjusted on tumour size and the number of sampled nodes (95% CI)	1.13 (1.03–1.24)	1 (Reference class)	0.91 (0.84–0.98)	0.01/0.01

Footnote see Table 2a.
